# WGS Analysis and Interpretation in Clinical and Public Health Microbiology Laboratories: What Are the Requirements and How Do Existing Tools Compare?

**DOI:** 10.3390/pathogens3020437

**Published:** 2014-06-11

**Authors:** Kelly L. Wyres, Thomas C. Conway, Saurabh Garg, Carlos Queiroz, Matthias Reumann, Kathryn Holt, Laura I. Rusu

**Affiliations:** 1 IBM Research—Australia, Level 5, 204 Lygon Street, Carlton, Victoria 3053, Australia; E-Mails: tconway@au1.ibm.com (T.C.C.); saurabh.kr.garg@gmail.com (S.G.); caxqueiroz@gmail.com (C.Q.); laurusu@au1.ibm.com (L.I.R.); 2 IBM Research—Zuerich, Säumerstrasse 4, Rüschlikon 8803, Switzerland; E-Mail: MRE@zurich.ibm.com; 3 Bio21 Institute, University of Melbourne, 30 Flemington Road, Melbourne, Parkville, VIC 3052, Australia; E-Mail: kholt@unimelb.edu.au

**Keywords:** genomics, microbiology, clinical, public health, bioinformatics

## Abstract

Recent advances in DNA sequencing technologies have the potential to transform the field of clinical and public health microbiology, and in the last few years numerous case studies have demonstrated successful applications in this context. Among other considerations, a lack of user-friendly data analysis and interpretation tools has been frequently cited as a major barrier to routine use of these techniques. Here we consider the requirements of microbiology laboratories for the analysis, clinical interpretation and management of bacterial whole-genome sequence (WGS) data. Then we discuss relevant, existing WGS analysis tools. We highlight many essential and useful features that are represented among existing tools, but find that no single tool fulfils all of the necessary requirements. We conclude that to fully realise the potential of WGS analyses for clinical and public health microbiology laboratories of all scales, we will need to develop tools specifically with the needs of these laboratories in mind.

## 1. Introduction

The recent advent of NGS (Next-Generation (DNA) Sequencing) technologies has revolutionised the field of bacterial genomics. It is now possible to generate whole-genome sequences (WGSs) for tens or hundreds of bacterial isolates on a single sequencing instrument in a matter of hours/days. Consequently, large numbers of genomes (*n* > 100, but the most recent examples comprise *n* > 1000) can be collected and compared within a single study, providing insights into the epidemiological and evolutionary dynamics of clinically important bacteria [1,2,3,4,5,6].

**Figure 1:**
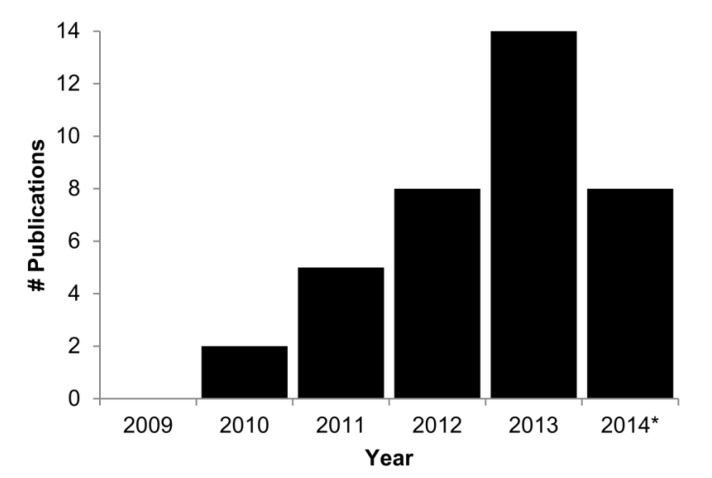
Publications describing clinical/public microbiology whole-genome sequence (WGS) analyses. Publications as listed in the PubMED database were identified by use of search terms “whole-genome” AND “outbreak”. Abstracts were manually screened to include only those describing bacterial outbreak investigations or evaluation of WGS analyses for outbreak investigations. * Publications as of 26 May 2014 only.

The advances which permit the investigation of large numbers of bacterial genomes also have the potential to transform the domain of clinical/public health microbiology [7,8,9,10,11]. Traditional bacterial characterisation comprises multiple species-specific phenotypic and molecular tests, requiring several days or even weeks for completion [8,12]. However, such characterisation can also be achieved through the analysis of WGS data. Indeed, over the past few years increasing numbers of studies have demonstrated the feasibility of using WGS data for clinical/public health microbiology purposes (Figure 1, e.g., [13,14,15,16,17,18]).

Species identification is a crucial step in any clinical/public health microbiology investigation and has traditionally been achieved through a combination of culture, microscopy and biochemical assay techniques. More recently, MALDI-TOF MS (matrix-assisted laser desorption ionization time of flight mass spectrometry) species identification techniques have been developed and successfully applied to purified samples cultured on solid agar medium [12]. NGS technologies, as have generally been applied in the clinical/public health microbiology context, also follow culture and purification of the infectious organism. However, culture-independent NGS can potentially be used to diagnose bacterial infections via deep sequencing of 16S rRNA amplicons or whole genomic DNA, but such applications are still in the early research phases [19]. Consequently, given the speed and low cost of MALDI-TOF MS, NGS is unlikely to replace this technique for species identification in the near future, but rather replace subsequent characterisation procedures through WGS of purified isolates [7,8,20].

Individual gene sequences for classification by traditional genotyping schemes e.g., multilocus sequence typing (MLST) can be extracted from WGS [14,16,21]. Such protocols have been successfully applied to inform epidemiological investigations and allow backwards compatibility with existing data. Investigation of disease outbreaks, requiring higher discriminatory power than that provided by traditional genotyping methods, can be achieved through comparison of variation across the wider genome [13,14,16,17,18]. Virulence and antibiotic-resistance phenotypes can also be estimated from WGS by extraction of virulence/toxin- and antibiotic-resistance gene sequences [14,15,22]. In the latter case the utility of WGS is limited by our knowledge about the relationships between genotypes and phenotypes. As such, WGS can indicate antibiotic-resistance by identification of known resistance-conferring mutations or genes, but cannot reliably indicate susceptibility when unknown or novel resistance mechanisms may be present. Unlike traditional epidemiological typing techniques, traditional antibiotic-susceptibility testing techniques are typically inexpensive and fairly fast (18–24 h following initial culture) [7]. For these reasons WGS is unlikely to completely replace traditional antibiotic-susceptibility testing. Nevertheless, rapid indication of resistance is a valuable source of information for clinicians and can be obtained at next to no extra cost when WGS data are generated for epidemiological purposes anyway.

Unfortunately, a number of challenges exist that restrict the use of WGS for clinical/public health microbiology investigations [11]. One of the most significant barriers to routine WGS use is the lack of user-friendly and automated analysis software [7,9,13,18,20]. Analyses of WGS data can be complex, requiring specialist knowledge and bioinformatics skills for execution of multiple sequential analysis components (e.g., as described in [14]). Furthermore, analysis execution, data-management and meaningful interpretation of results will be especially challenging in a high-throughput laboratory setting, where hundreds of isolates must be characterised and compared [8].

Analysis complexity is a difficulty common to both microbiology and human clinical WGS analyses, but other problems are unique to microbiology investigations. Firstly, comparative analyses of data representing multiple independent individuals distributed through time and across geographic jurisdictions will be a regular requirement for microbiology laboratories. As such analysis protocols or workflows must be standardised, fully repeatable and easily shared between laboratories [20]. Secondly, microbiology laboratories must characterise isolates of a large number of different species, each of which may require a different set of tests.

This review follows those describing the potential uses of, challenges and impact of WGS technologies for clinical/public health microbiology laboratories [7,8,11,20]. We believe that to realise this impact, laboratory staff will require access to tools providing: (1) intuitive and automated high-throughput analysis execution; (2) management and meaningful presentation of results; and (3) automated record-keeping. Here we present an overview of these requirements followed by a discussion of the relevant features, benefits and constraints of a number of existing WGS analysis execution tools. Most of these tools were not specifically designed for use in clinical/public health laboratory settings, rather to provide flexible solutions for research purposes. Nevertheless we hope this discussion will inform and inspire clinical/public health microbiologists, bioinformaticians and computer scientists alike, such that we can work together to develop the tools that will make routine WGS use a reality.

## 2. What Types of Analyses Are Required?

To appreciate the relevant features of WGS analysis tools we must understand the specific types of analyses that are required for clinical/public health microbiology investigations (hereafter simply called “microbiology investigations,” see Table 1).

NGS technologies generate large volumes of sequence data comprising short, overlapping sequence reads (generally thousands to millions of reads each between 100 and 3000 bp long for the current versions of popular sequencing machines). Each error free read represents a small fragment of the true genome (including the bacterial chromosome and any phage or plasmid sequences present within the cell). To make sense of this sea of sequences, a large number of processing and analysis steps are required.

In general there are two analysis strategies; the first comprises reference-based read mapping and identification of variant sites relative to a reference sequence. In this strategy each short sequence read is individually aligned against a longer reference sequence. The reference sequence may be a complete bacterial genome [14,17], a collection of representative gene sequences e.g., resistance or toxin gene sequences [14], or an entire database of known epidemiological typing locus variants [14,23].

The second analysis strategy comprises *de novo* assembly of sequence reads into longer contiguous sequences, subsequent extraction, alignment and comparison of regions/genes of interest. Through this technique comparison of antibiotic-resistance, toxin and epidemiological typing gene content/sequence variation is achieved [24,25]. Comparison of variation across the wider genome is possible using an extension of the MLST technique [21], whereby a set of key genomic loci are defined and characterised for each bacterial isolate. Subsequently, isolate genomes may be compared by variation at these loci [16].

An important component of microbiology investigations is the estimation of evolutionary relationships between isolates. These estimates allow microbiologists to track spatiotemporal changes in bacterial populations, to identify disease outbreak strains, transmission routes and outbreak sources.

Evolutionary relationships can be estimated through construction of phylogenetic trees or networks, and there are many methods through which this can be achieved. The inputs for these analyses can be derived from the results of either reference-based mapping or *de novo* assembly. In the latter case homologous sequence regions must be aligned, either through concatenated alignment of individual genes or through alignment of complete genomes.

The phylogenetic method best-suited to a particular investigation will likely depend on the characteristics of the sample population and the question of interest e.g., understanding the evolutionary relationships among a diverse set of isolates collected over several years *vs.* understanding the relationships among a group of closely related outbreak isolates. An important factor to consider before conducting a phylogenetic analysis is the effect of genetic recombination upon the sample population. Some bacterial species are highly recombinogenic *i.e.*, segments of DNA are frequently transferred between individuals through mechanisms other than ancestral inheritance, whilst other species rarely undergo recombination. High rates of recombination can obscure the phylogenetic signal within a data sample and must be taken into account by the use of an appropriate phylogenetic method or by prior exclusion of any affected genomic regions.

**Table 1:** Analytical capability of targeted Next-Generation (DNA) Sequencing (NGS) analysis solutions with respect to clinical and public health microbiology investigative methods.

Solution	Date of Publication/First Release	Upload/Analyse Raw Sequence Data	Reference-Based Mapping	*de novo* Assembly	Variant Calling	Typing analyses (e.g., MLST)	Comparative Typing Analyses	Multiple Sequence Alignment	Phylogenetic Tree/Network Construction
**Generic NGS Analysis Solutions:**									
BioNumerics ^a^	1992	Yes	Yes	Yes	Yes	No ^c^	Yes	Yes	Yes
CLC Genomics Workbench ^a^	2008	Yes	Yes	Yes	Yes	No	No	Yes	Yes
Galaxy ^b^	2007	Yes	Yes	Yes	Yes	No	No	Yes	Yes
**Specific Bacterial NGS Analysis Solutions:**									
BIGSdb	2010	No	No	No	Yes	Yes	Yes	Yes	Yes
Center for Genomic Epidemiology Web Portal	2011	Yes	No	Yes	Yes	Yes	Yes	No ^d^	Yes
Ridom SeqSphere+ ^a^	2013	Yes	Yes	Yes	Yes	Yes	Yes	Yes	Yes
snp-search	2013	No	No	No	No	No	Yes	No ^e^	Yes

^a^ Commercial product; ^b^ Features/analyses described for the default installation and tools available in the Galaxy Tool Shed as of September 2013 only; ^c^ There is a function to automate typing analyses from first generation but not next-generation sequence data; ^d^ The Center for Genomic Epidemiology snpTree tool deals only with variant call information and thus multiple sequence alignment *sensu stricto* is not applicable; however the tool will generate a pseudo-alignment of concatenated single nucleotide polymorphisms; ^e^ as described for snpTree, snp-search deals only with variant call information but will output a pseudo-alignment of concatenated single nucleotide polymorphisms in FASTA format.

Another important consideration is that of core and accessory genome regions, the former shared by all members of a species, the latter variably present within a species e.g., antibiotic-resistance genes. When performing phylogeny construction it is important to consider which regions of the genome are universally present among the specific isolates of interest. Inclusion of variably present regions may bias the analysis and obscure true phylogenetic relationships, whilst unnecessary exclusion of a conserved region may reduce resolution.

In general, both read mapping and *de novo* assembly of a single bacterial genome can be completed in under one hour (estimates specifically for an *Escherichia coli* genome at 30× read depth are 15–20 min and 10–20 min, respectively [26]). Analyses of MLST or resistance gene sequences may take only a matter of seconds (when a small number of loci are extracted from pre-assembled genomes) or up to an hour (when reference-based mapping is used to search for a large number of genes and/or variants). The time required for phylogenetic inference is highly dependent on the choice of algorithm and the number of independent isolates to be included, ranging from minutes to several hours. Error rates vary by the choice of analysis algorithm and whether the algorithm is well suited to the sequence data in question (dependent on sequencing technology). Error rates can be controlled by appropriate analysis parameter selection and quality control procedures e.g., minimum read depth requirements. Readers interested in learning further details about bacterial WGS data analyses are directed to [27]. For overviews of reference-based read mapping and *de novo* assembly algorithms see [28] and [29], respectively.

The most appropriate WGS analysis strategy for microbiology investigations remains a subject of debate. Whilst the majority of previous poof-of-concept studies have favoured reference-based mapping techniques (see Table 2 for examples), to our knowledge there has not yet been a thorough conclusive study to evaluate and compare the accuracy, efficiency and cost-effectiveness of the two primary strategies described here. In fact it may be the case that neither strategy is optimal across all types of investigations or for all species *i.e.*, due to differences in genomic variability (core *vs.* accessory genome size and recombination rates). A detailed discussion about the merits of these strategies or alternative strategies is beyond the scope of this review.

## 3. Computational Requirements in the Multi-Core, Multi-Processor Era

With the reduction in sequencing cost and time, the bottleneck in WGS is shifting to computation. Challenges of data processing, integration, analysis, storage and display limit our use of genomic information [20]. State of the art personal computers contain fast processors with multiple processing cores but will not meet the requirements of high-throughput, multi-user laboratories. Computing clusters comprise multi-core processors linked via a communication network and provide a useful alternative. Indeed, the life science community increasingly accesses supercomputing facilities with tens of thousands of such processors.

While multiple cores on single processor computers have physical access to the same memory (shared memory), clusters and supercomputers are parallel systems (both their processing power and memory are physically separated). This poses a problem for many computationally intensive algorithm implementations [30], which are often built for shared memory computers. Since algorithms often go through extensive repetitive loops, the implementation of parallelism in shared memory is straightforward. To leverage the computing power of distributed memory systems, algorithms often need to be re-engineered, a process which has not been readily applied to current tools.

Given that the memory of a processor can hold a reference sequence, sequence reads can easily be divided by the number of processors available such that read mapping is carried out in a parallel fashion, scalable to thousands of processors [31]. Unfortunately, *de novo* assembly is a much harder task to parallelise [32] since the graph that needs to be built to assemble a whole-genome becomes very large. Subsequently, graph traversal and sequence determination are computationally expensive. Parallel computing in graph theory is an established field and the first attempts to implement *de novo* assembly approaches on distributed memory computers do show potential, though more work is required [32].

Scalability of algorithms in terms of computation and memory is particularly important for comparative analyses [30]. The alignment of whole-genomes, for example, can take hours or days. It is evident that the computational efficiency of such tasks needs to be improved to allow fast alignment of hundreds of genomes, such as required for microbiology investigations. Little effort is seen in this area but breaking this barrier will yield much-needed advances in making feasible the use of WGS in microbiology laboratories.

We will not discuss further details of the computational requirements of WGS data analyses, but it is worth considering that the ability of a tool or platform to enable parallel data processing will be an advantage in high-throughput laboratory settings.

## 4. Other Requirements and Considerations

Management of results and data files are also important considerations for the use of WGS data in microbiology investigations. It is imperative that sequence data and results can be linked correctly to bacterial isolate identifiers and that appropriate data are used for comparisons. Without these links the data cannot be interpreted meaningfully and the conclusions may be misleading, which is particularly concerning given the potential impact for clinical and public health decision-making.

Maintenance of accurate analysis records (algorithms, parameters and results) will be essential. These records, along with those detailing the dates of the analyses and the identities of the staff members responsible, should form a complete audit trail compliant with clinical regulatory requirements. Similarly, any analysis tools must adhere to data privacy and security regulations [20]. Finally, analysis workflows must be standardised, repeatable and easily shared between laboratories [20].

## 5. Current Techniques for Bacterial WGS Analyses

Previous microbiology investigations were conducted by, or in consultation with, specialist bioinformaticians and largely relied upon the use of individual command-line tools. Sequential and/or repetitive analysis steps were manually executed or combined through the use of custom scripts. The methodologies and analysis execution techniques of 10 recent outbreak investigations are summarised in Table 2. For the non-bioinformatics specialist (such as many microbiology laboratory staff) the execution of these analyses may be a daunting prospect. However, there are a number of targeted solutions which aim to simplify this process. A comparison of the analytical capabilities of these solutions is shown in Table 1, while specific features of interest are highlighted in the text below.

**Table 2:** Methodologies and analysis execution techniques of recently published bacterial outbreak investigations.

Species	Focus ^a^	Type(s) of Methods	Tools ^b^	Ref.
*Clostridium difficile/Escherichia coli*	**Transmission**	Mapping, variant calling, phylogeny	Independent and/or custom	[15]
	**MLST**	*de novo* assembly, sequence search	Independent and/or custom	
	**Antibiotic resistance genes**	*de novo* assembly, sequence search	Independent and/or custom	
*E. coli*	**Diversity/Resolution**	Mapping, variant calling, phylogeny	Independent and/or custom	[33]
	**Relationship to historical isolates**	*de novo* assembly, multiple sequence alignment	Independent and/or custom	
	**Gene content comparison**	*de novo* assembly, annotation	Independent and/or custom	
*E. coli*	**Differentiation**	Mapping, variant calling	CLC Genomics Workbench	[34]
	**MLST**	*de novo* assembly, sequence search	CLC Genomics Workbench	
	**Antibiotic resistance genes**	*de novo* assembly, sequence search	CLC Genomics Workbench/custom	
	**Relationship to historical isolates**	Mapping, variant calling, network/*de novo* assembly	CLC Genomics Workbench/custom	
*Klebsiella pneumoniae*	**Transmission**	Mapping, variant calling, minimum spanning tree	Independent and/or custom	[17]
*Legionella pneumophilia*	**Differentiation**	Mapping, variant calling, phylogeny	Independent and/or custom	[35]
*Mycobacterium tuberculosis*	**Diversity/Resolution**	Mapping, variant calling, phylogeny	Independent and/or custom	[36]
*Neisseria meningitidis*	**Diversity/Resolution**	*de novo* assembly, sequence search	Independent/BIGSdb	[16]
	**MLST, other typing**	*de novo* assembly, sequence search	Independent/BIGSdb	
*Shigella sonnei*	**Differentiation**	Mapping, variant calling, phylogeny	Independent and/or custom	[37]
*Staphylococcus aureus*	**Differentiation**	Mapping, variant calling, multiple sequence alignment (references), phylogeny	Independent and/or custom	[13]
*S. aureus*	**Differentiation**	Mapping, variant calling, phylogeny	Independent and/or custom	[14]
	**MLST**	Mapping, read depth analysis, variant calling	Independent and/or custom	
	**Toxin genes**	Mapping, read depth analysis, variant calling	Independent and/or custom	
	**Antibiotic resistance genes**	Mapping, read depth analysis, variant calling	Independent and/or custom	

^a^ Differentiation—the aim was to differentiate outbreak from non-outbreak strains; Diversity/resolution—the aim was to assess the diversity and/or resolve the phylogenetic relationships among outbreak strains; Transmission—the aim of the study was to resolve the relationships between strains specifically to enable transmission tracing; ^b^ Independent and/or custom—refers to the use of independent bioinformatics tools for different analysis stages (e.g., separate open source tools for mapping and variant calling) and/or custom analyses/scripts.

## 6. Generic NGS Analysis Solutions

### 6.1. BioNumerics

BioNumerics (including Kodon, Applied Maths NV, Sint-Martens-Latem, Belgium) is a software package providing analysis capabilities for a range of biological data, including a number of traditional bacterial typing techniques, antibiotic-resistance profiling and NGS data processing/analyses. A particularly useful feature is the ability to link with international bacterial typing databases (e.g., online MLST databases) in a way that enables automated processing and genotype designation for traditional data types (e.g., Sanger sequence chromatograms). BioNumerics will highlight ambiguities in the data, facilitating easy and time-efficient correction through the graphical user interface (GUI). However, BioNumerics does not currently provide the facility to derive typing or antibiotic-resistance gene information directly from WGS data.

Current WGS data analyses include read quality control, *de novo* assembly, reference-based mapping, variant calling, multiple genome alignment, structural variation analysis and phylogeny construction. Data from a range of NGS platforms can be managed and analysed in single or batch mode, as would be required in a high-throughput environment. Analysis pipeline templates can also be created in the GUI, saved and used to assist execution of repetitive tasks.

Sequence read files are treated as database objects that can be linked to additional user-defined database entries e.g., isolate metadata. Data can be screened by database fields such that information, data and analyses representing groups of interest can be easily identified; a feature facilitating rapid, appropriate selection of data for comparative analyses and interpretation of results. BioNumerics also includes comprehensive audit trail capabilities, designed to comply with regulatory standards.

### 6.2. CLC Genomics Workbench

The CLC Genomics Workbench (CLC bio, Aarhus, Denmark) is a genomics analysis application, providing an “out-of-the-box” solution for NGS analysis. Several recent bacterial genomics articles have demonstrated the use of this application [22,38,39,40,41], which supports data from all major sequencing platforms and allows the user to execute a range of custom-built analyses through a GUI. The application includes general DNA sequence analysis tools (e.g., sequence manipulation, multiple-sequence alignment, phylogeny construction) and NGS-specific analysis tools (e.g., reference-based read mapping and *de novo* assembly). Analyses may be executed for individual read/read-pair sets or for read set batches. The tool also allows results to be visualised, saved and printed.

Users can construct analysis workflows with the drag-and-drop workflow tool, thereby automating the execution of sequential analysis components. Such workflows reduce the labour intensity of repetitive tasks without the need for programming or scripting knowledge. Analysis modules may be selected, arranged and connected on the workflow canvas. Appropriate input and output file types are indicated and connection of incompatible modules is prevented by the automatic workflow validation function. However, in its current form, the CLC Genomics Workbench workflow tool cannot include comparative analyses such as multiple-sequence alignments or phylogeny construction, meaning that such analyses must be executed independently whenever they are required. This caveat is particularly important in the context of microbiology laboratories, where frequent comparative analyses are required.

With respect to data-management and record-keeping, the CLC Genomics Workbench includes a function to allow the user to link metadata with raw NGS data files. A search function allows searching over sequence data files by metadata attributes. The “history” function maintains a record for all files, indicating the analyses (and the associated parameters) applied to, or resulting in the generation of the file. Unfortunately though, there is no function to search History records.

### 6.3. Galaxy

Galaxy is a free web-based NGS data analysis tool, primarily developed by researchers at the Pennsylvania State and Emory Universities, USA [42]. This tool integrates a number of NGS analysis modules/algorithms including those for sequence read quality assessment/filtering, reference-based mapping, variant calling and *de novo* assembly. The Galaxy project provides an open Galaxy installation where users can create and save data libraries, analysis workflows and analysis outputs. Alternatively, users can install and use Galaxy locally on their own environment. Analysis modules additional to those available through the default installation may be obtained from the Galaxy Tool Shed, an online repository where the Galaxy development team and users can share modules and/or “wrappers” (components required for, and enabling the integration of Galaxy with other tools).

Galaxy workflows are generated by selection and configuration of analysis components, which consists of a series of drop-down menus and/or text input boxes for input data file and parameter specification. If required and provided by the appropriate “wrapper” developer, users can view written descriptions of analyses and their associated parameters. During workflow construction, data selection is restricted to those files representing compatible data formats. As such, connection of incompatible analysis components is prevented.

Workflows may be saved, edited or run at any time, allowing easy completion of repetitive analysis protocols. However, it is not possible to select an analysis workflow designed for a single input file and apply to a batch of files simultaneously. Similarly, in the default configuration it is not possible to design a single analysis workflow which will perform sequential analyses on multiple input files and subsequently combine the outputs of these analyses into a single comparative analysis e.g., as would be required to map multiple sets of sequence reads against a reference genome, call variants and compare the variants across all read sets. This caveat would likely be a major draw-back for any tool implemented in a high-throughput setting and has likely contributed to the lack of reported use of Galaxy for large-scale bacterial genomics investigations (though use for partial, small-scale bacterial genomics analyses has been reported [43,44]).

Galaxy maintains a “history” of all analyses, their associated input/output files and parameters, providing a record of the precise processes from which results are derived without the need for users to maintain their own notes.

Unlike the CLC Genomics Workbench, the Galaxy source code is freely available, allowing full assessment by the wider research community [45] and meaning that users possessing sufficient IT/bioinformatics skills can modify and/or extend the tool to meet their needs. For example, researchers at the University of Mainz, Germany developed an NGS laboratory information management system extension to the Galaxy platform [46]. The tool, designed for laboratories at which sequence data is generated, provides a mechanism to track the status of DNA samples submitted for NGS.

### 6.4. Tools from Sequencing Technology Vendors

Another solution is the Illumina BaseSpace cloud service (Illumina Inc., San Diego, CA, USA), launched late 2012. In November 2012 an additional BaseSpace Apps service was launched to provide users with a range of data analysis tools. Apps, developed by Illumina and collaborators, provide several analysis capabilities including *de novo* assembly of bacterial genomes. Illumina HiSeq and MiSeq NGS machines can push data directly to BaseSpace, where it can be analysed in the cloud. Users can create personal accounts and choose whether their data should remain private or can be shared with others.

A similar offering is provided by Life Technologies (Carlsbad, CA, USA) for its Ion Torrent sequencer customers; the Torrent Suite software, providing read-mapping and variant calling capabilities among others is pre-installed on Ion Torrent sequencing machines. Users can also install software plugins to enable a range of additional analyses such as *de novo* assembly.

## 7. Specific Bacterial WGS Analysis Solutions

### 7.1. BIGSdb

BIGSdb (Bacterial Isolates Genome Sequence database) is a solution specifically designed for analysis of large numbers of bacterial genomes [47]. This web-based application allows users to construct a database of bacterial isolate records and associated metadata, each linked to an unlimited amount of isolate-specific sequence data (in pre-assembled format). Sequences can be automatically scanned and tagged for loci representing pre-defined or custom user-defined schemes e.g., MLST schemes. Sequences of tagged loci can be compared, exported in a variety of formats or analysed by SplitsTree4 [48] which produces a network representation of evolutionary relationships. The BIGSdb Genome Comparator tool allows allelic comparison of annotated coding sequences (CDS) across isolate genomes, and construction of neighbour-net trees from these data (CDS—as defined within reference genome annotations provided by the user or from the web-accessible Genbank database hosted by the National Centre for Biotechnology Information, NCBI).

Analysis of data through BIGSdb does not require advanced bioinformatics skills. However, the application only supports assembled sequence data and does not provide the tools required to produce these assemblies. Nevertheless, BIGSdb has been successfully employed for both small and large scale bacterial genomics analyses [16,40,49,50,51].

As mentioned above BIGSdb allows the user to link sequence data with isolate identifiers and metadata. Following analysis, allelic assignments for any number of defined loci can be automatically appended to isolate records, ensuring that these results are linked to the correct isolates. However, BIGSdb does not maintain user-accessible logs or records of analyses completed through the application.

### 7.2. Center for Genomic Epidemiology Web Portal

Researchers at the Center for Genomic Epidemiology in Denmark have developed a suite of tools specifically for analysis of bacterial WGS. These tools are available at www.genomicepidemiology.org and have been applied to investigations of the use of WGS for bacterial surveillance [52,53]. The tools include those to determine multi-locus sequence types [25], identify acquired resistance or virulence genes [54] and construct genome-wide phylogenies [55].

In the case of MLST and resistance gene identification, users are required to select a single file or pair of files containing the desired input data. These data should be in either complete/draft assembly FASTA format (single file) or raw sequence read format (single or pair of files). MLST locus sequences and those of acquired antibiotic-resistance genes are identified by BLAST search over assembly contigs (generated automatically by the tools when provided raw sequence reads by the user). A drop down menu is used to specify the type of input file (assembly contigs *vs.* raw reads, in the latter case the sequencing technology must be specified) and the desired MLST scheme or percentage identify BLAST match threshold for resistance gene identification. Results are displayed in table format. These tools require no specialist bioinformatics skills, pre-processing of sequence reads or specialist computing resources and return results in an easy to interpret format e.g., relevant antibiotic resistance phenotypes are indicated automatically upon identification of resistance genes and MLST locus sequences are automatically queried in the appropriate database, returning both allele and sequence type assignments. However, users cannot execute the tools in batch mode meaning that analyses must be selected/submitted independently for each individual isolate, a highly repetitive task for high-throughput laboratories. Furthermore, users must manually keep track of sequence data files, results and the isolates to which they pertain.

The Center for Genomic Epidemiology currently provides two genome-wide phylogenetic methods, the first, known as snpTree, utilises reference-based mapping and variant calling [55], the second, known as NDtree, utilises a custom nucleotide distance method [53]. In both cases users must select sequence read files representing the group of isolates to be analysed. Analysis parameters and reference sequences can be selected by the drop-down menus as appropriate. Upon completion of the analysis a graphic representation of the phylogeny is shown in the web portal. Output files are also available for download and include phylogeny images, variant call files and a range of SNP summaries (snpTree). Polymorphisms identified during the snpTree analysis can also be downloaded in a format compatible with stand-alone tree drawing tools. Production of genome-wide phylogenies is a multi-step process, but through the use of the Center for Genomic Epidemiology tools it can be achieved with minimal user-machine interaction. Unfortunately though, neither of these phylogeny tools can account for genetic recombination among the sample genomes. Furthermore, users must again maintain their own records of data files, analyses and results.

### 7.3. Ridom SeqSphere+

Ridom SeqSphere+ is an offering from Ridom GmbH (Münster, Germany) [56]. Like BIGSdb, Ridom SeqSphere+ provides users with the capability to analyse bacterial WGS data through an extension of the MLST method (here called MLST+). Users can create projects and analysis schemes, upload isolate metadata and sequence assembly data, analyse the data and visualise the results. Sequence assemblies are linked to user-defined isolate metadata and analysis results (allele assignments). Allele sequences can be concatenated and aligned within the software or exported for use with other tools. Users can search isolates across metadata fields and select isolates for inclusion in comparative analyses (e.g., generation and visualisation of distance matrices and minimum spanning trees)—such a feature will likely be indispensable for ensuring efficient and appropriate isolate comparisons.

The latest version of Ridom SeqSphere+ allows upload of raw sequence reads which are subsequently assembled *de novo* or mapped to a reference. The user has the option of uploading raw reads, raw sequence assemblies or assemblies plus read-mapping information (where sequence reads have been mapped back to the consensus assembly contigs). In the latter case, the software can incorporate the additional information for quality control and error correction procedures. Users can download predefined MLST analysis schemas or use the inbuilt target generator tool to define MLST+ schemas. This tool identifies conserved, candidate loci across a user-defined set of reference genomes.

In addition to its analysis capabilities, Ridom SeqSphere+ provides record-keeping and audit tracking capabilities. As described, sequence data and results can be linked to isolate metadata, although users do need to choose to save results and manually specify the file directory location. A log of user actions including the execution of analyses and data editing (e.g., manual data corrections) is also maintained.

### 7.4. Snp-Search

Researchers from the Microbiology Services at Public Health England have developed a SNP (single nucleotide polymorphism) data management and manipulation tool known as snp-search [57]. Given a reference sequence annotation and a set of input variant call format files (representing variant positions identified among sequence reads mapped to a given reference sequence) the tool can build a searchable SNP database within which bacterial isolate identifiers are linked to their representative sequence variants. Variants are contextualised using the reference sequence annotation, groups of SNPs unique to or differing among a given isolate set can be identified and phylogenies inferred. SNPs may be excluded from phylogenetic analyses at the preference of the user e.g., those below a given quality threshold or associated with mobile genetic elements (the latter of which are part of the accessory genome).

The ability to manage, search and analyse WGS data by isolate identifiers, and in the context of reference sequence annotations is key for microbiology investigations. However, snp-search does not have complete record-keeping or audit trail functions and its use is dependent on the ability to generate read-mapping data from raw sequence reads.

## 8. Workflow Management Systems

Numerous workflow management systems (WMS, see Table 3 and Table 4) have been developed to simplify the execution of bioinformatics protocols. These systems allow users to build, save and execute custom workflows comprising the desired analysis components, thereby allowing the automation of sequential analysis execution. WMS can be divided into those that were specifically designed for molecular biology or DNA sequence analysis purposes and those that were designed for generic *in silico* analytics tasks (Table 3). For example, Wildfire [58] provides a GUI designed to create bioinformatics workflows using tools such as those provided in EMBOSS (The European Molecular Biology Open Software Suite) [59]. EMBOSS tools are preconfigured such that parameters can be specified through the template form drop-down menus. Additionally, users with adequate bioinformatics skills may integrate third-party locally installed analysis tools; however web-based services are not supported. Tools representing the independent stages in the workflow can be arranged and connected in a drag-and-drop workflow canvas. Workflow execution and progress monitoring are also achieved through the GUI.

**Table 3:** Workflow management systems: general features.

Application	Native App/Web-based	Security/Authentication Available?	Workflow Design	Analysis Progress Monitoring	Ability to Stop/Pause/Resume Workflows?	Rerun Workflows with Different Parameters?
Anduril	Native	Unclear	AndurilScript	Yes	Stop only	Yes
Biomolecular Hub	Native	Yes	Drag-and-drop	Yes	Stop only	Unclear
Chipster	Both	Yes	List	Yes	No	Yes
NG6	Web-based	Yes	Drag-and-drop	Yes	No	No
VIBE	Client/server	Yes	Drag-and-drop	Yes	Stop only	No
Wildfire	Native	No	Drag-and-drop	Yes	No	Yes
Kepler	Native	No	Drag-and-drop	Yes	Yes	Yes
KNIME	Both	Yes	Drag-and-drop	Yes	Yes	Yes
Pegasus	Native	No	Dax files	Yes	Stop only	Yes
Pipeline Pilot	Native	Yes	Drag-and-drop	Yes	Yes	Yes
Platform Process Manager (PPM)	Native	Yes	Drag-and-drop	Yes	Yes	Yes
Taverna	Native	Yes	Drag-and-drop	Yes	Yes	Yes

**Table 4:** Workflow management systems: analysis-specific features and references. API—Application Programming Interface.

Product	Generic *vs* Genetic Analysis-Specific	NGS Analysis components?	Integration of third-party analyses?	Languages for Integration of New Tools	Reference and/or Vendor
Anduril	Specific	Limited	Yes	XML	[60]
Biomolecular Hub	Specific	No	Unclear	Java	IDBS, Guildford, UK
Chipster	Specific	Limited	Yes	R/configuration changes	[61]
NG6	Specific	Yes	Yes	Python (through XML)/Smarty template	[62]
VIBE	Specific	No	Yes	XML/Java (through API)	INCOGEN Inc., Williamsburg, VA, USA
Wildfire	Specific	No	Yes	Extended ACD syntax (through API)	[58]
Kepler	Generic	No	Yes	Java	[63]
KNIME	Generic	No	Yes	Eclipse Plugins	KNIME.com AG, Zurich, Switzerland
Pegasus	Generic	No	Yes	DAX workflow definitions	[64]
Pipeline Pilot	Generic	Yes	Yes	Java/Perl/VBScript/SOAP	Accelrys, Inc., San Diego, CA, USA
Platform Process Manager (PPM)	Generic	No	Yes	Custom scripts	IBM Corp., Armonk, NY, USA
Taverna	Generic	Yes	Yes	SOAP/template library	[65]

Chipster [61] is an analysis platform and WMS specifically designed for high-throughput molecular biology analyses. Chipster includes a number of WGS analysis tools related to sequence read quality control, mapping and variant calling, but does not contain tools required for *de novo* assembly. Users with adequate skills may integrate third-party analysis components, e.g., those required for *de novo* assembly. Chipster allows users to import data, select, configure and execute analyses, and generate custom workflows through a GUI. An interesting feature is the ability to label data by project or experimental treatment, such that the interpretation of analysis results is simplified e.g., for genotype-phenotype association investigations. It is not clear however, how this feature may be applied to assist clinical or epidemiological studies.

Taverna [65] is a popular, generic WMS which allows users to design workflows comprising distributed web-based tools e.g., those provided by NCBI and EBI (European Bioinformatics Institute). As described for the WMS mentioned above, users with adequate bioinformatics skills may also integrate third-party tools. Users can compose their own workflows through the GUI drag-and-drop canvas or may search for and utilise workflows shared in the myExperiment web repository [66]. Workflows can be designed such that batches of sequence read data can be processed simultaneously and the results integrated for downstream analyses. As mentioned previously, such a feature is necessary if sequence reads representing multiple different bacterial isolates are to be processed and the results compared.

Other generic WMS for which molecular biology and/or WGS analysis tools have been integrated (Table 3 and Table 4) include the IDBS Biomolecular Hub (ID Business Solutions, Guildford, UK) and Pipeline Pilot (Accelrys Inc., San Diego, CA, USA). In the latter case, more than 100 DNA sequence analysis tools are available and NGS-specific analysis components are under development in collaboration with Oxford Nanopore Technologies Ltd (the developers of a new nanopore-based NGS technology, based in Oxford, UK). The Pipeline Pilot NGS analysis capabilities include sequence read quality control, read mapping, variant calling and *de novo* assembly.

Other useful WMS features include the ability to monitor analysis progress, pause/resume/stop workflows, and re-run workflows with different parameter values (Table 3). Many WMS also provide the ability to highlight or correct workflow errors during design or run-time e.g., Wildfire uses automated annotations on the workflow canvas to alert users to errors. The VIBE (INCOGEN Inc., Williamsburg, VA, USA) GUI will alert users to incompatibilities between analysis components e.g., when the output format of a component is not compatible with the input format of its downstream components. Additionally, most WMS allow analyses to be executed using some form of parallel processing. This type of processing can dramatically reduce the time required to complete analysis workflows and will be essential for completion of repetitive analyses in microbiology laboratories e.g., sequence read mapping for data representing multiple independent bacterial isolates.

With regards to data management, many WMS have little to no functionality. However, the IDBS Biomolecular Hub and NG6 [62] do provide such capabilities. The Biomolecular Hub enables the integration of multiple types of data in multiple formats and there is also an option to integrate data from public databases, a feature which is particularly useful for comparison of epidemiological typing results to those of previously characterised bacterial isolates. NG6 allows sequence data to be grouped by project and associated to relevant metadata [62].

Specific record-keeping functions are also absent from many WMS, although some allow a record of completed analysis workflows and the associated parameters, input/output file types to be saved. NG6 maintains a list of all analyses applied to each sequence data file and VIBE allows workflows to be saved in both template and archive form, whereby the former contains information required to execute the workflow anew, and the latter contains a record of the parameters, inputs, outputs and results of a completed workflow run. Unfortunately, within the latter WMS it is not possible for the user to search over their previous analysis histories. Should a user wish to return to an analysis record and review parameters, inputs or output file names for example, they will need to know the name of the analysis run of interest and the location of the relevant file. Such features may be adequate for short-term or low-throughput record-keeping requirements but will likely be inadequate for microbiology laboratories which must adhere to regulatory requirements.

## 9. Conclusions

Recent technological advances have moved us a step closer to the routine-use of WGS in microbiology laboratories, but the lack of automated and intuitive analysis tools remains one of the major barriers. In their recent review, Fricke and Rasko described the bioinformatic challenges associated with the application of bacterial WGS data in clinical settings, highlighting the need for standardisation, appropriate management of computing resources, data integration and storage [20]. Here we explored the specific requirements for microbiology investigations in further detail and discussed relevant features of a number of existing WGS data analysis solutions. Most WGS-specific solutions, including generic and those designed for specific bacterial WGS analyses, provide a user-friendly GUI through which analyses can be executed without advanced bioinformatics skills, a key requirement given that not all microbiology laboratories have access to bioinformatics specialists [7]. Most of these solutions also allow users to develop analysis workflows/pipelines within the GUI and/or allow easy execution of batch analyses, both of which will be essential in high-throughput laboratories. However, none of these solutions currently includes the complete set of analysis tools required by microbiology laboratory staff e.g., for automated derivation of epidemiological typing data, antibiotic-resistance information *and* appropriate phylogenetic inference for all necessary bacterial species.

WMS provide flexible analysis pipelining and execution solutions, most of which allow integration of third-party tools and thus can be configured to include all necessary analyses. An important caveat however, is that tool integration usually requires advanced bioinformatics skills. Additionally, whilst flexibility does permit the integration of all required analysis algorithms, routine use of WGS in clinical/public health settings will require standardisation. Perhaps then WMS could play a role if bioinformatics specialists were to develop standard workflows in consultation with public health professionals? However, data-management and record-keeping difficulties would remain, since most WMS provide little relevant functionality. In contrast, the WGS-specific solutions demonstrate many useful and essential features, such as the ability to link sequence data/results with isolate metadata, search over isolate metadata and maintain accurate records of all results/user actions. The latter of which is particularly important when seeking accreditation for clinical use.

As we reach the dawn of the new WGS era for clinical and public health microbiology, the need for appropriate data analysis solutions is well recognised. The tools discussed here can provide a partial or first pass solution, but it is clear that public-health, microbiology, bioinformatics and computer scientist specialists will now need to work together to develop complete solutions catering for microbiology laboratory needs. Only then will the power of WGS technologies be truly realised in this domain.

(Please note, the information described in this article, including existing tool functionality, was primarily collected by the authors during the year 2013, from publicly available sources. Every effort was made to ensure the accuracy of the information to our knowledge at this time.)
